# The Need to Develop an Individualized Intervention for Mathematics Anxiety

**DOI:** 10.3389/fpsyg.2021.723289

**Published:** 2021-10-20

**Authors:** Ahmed A. Moustafa, Ahmed A. Al-Emadi, Ahmed M. Megreya

**Affiliations:** ^1^School of Psychology, Western Sydney University, Sydney, NSW, Australia; ^2^Department of Human Anatomy and Physiology, The Faculty of Health Sciences, University of Johannesburg, Johannesburg, South Africa; ^3^Department of Psychological Sciences, College of Education, Qatar University, Doha, Qatar

**Keywords:** mathematics anxiety, intervention, cognitive behavioral therapy, math education, individualized treatment

## Introduction

Mathematics anxiety is prevalent in many societies around the world, ranging from 30 to 70% (for a review, see Betz, 1978; Dowker et al., 2016). More than 30% of 15-year-old students across the Organization for Economic Co-operation and Development (OECD) countries reported having mathematics anxiety (OECD, 2013). Several studies have shown that mathematics anxiety is related to both mathematics performance and joining Science, Technology, Engineering, and Mathematics (STEM)-related careers (Moustafa et al., 2017, 2020; Zhang et al., 2019). While most prior studies on the link between mathematics anxiety and interest in STEM careers are correlational, it is plausible to assume that mathematics anxiety at early school years (Krinzinger et al., 2009) could have led to low interest in STEM careers later in life. Importantly, there are almost no existing approved interventions for mathematics anxiety. The purpose of this current opinion article is to highlight the need to develop an individualized intervention that targets factors leading to mathematics anxiety in students.

## Mathematics Anxiety

Dreger and Aiken (1957) and Gough (1954) coined the term mathematic anxiety to refer to a feeling of tension, apprehension, or even dread interfering with number manipulation and mathematical problem solving (Ashcraft and Faust, 1994; Ashcraft, 2002; Moustafa et al., 2017; Alkan, 2018; Lukowski et al., 2019a,b). Mathematics anxiety was found to be related to the complexity of mathematics activities (Faust et al., 1996; Namkung et al., 2019) and it is separate from other anxieties such as science anxiety (Megreya et al., 2021). Artemenko et al. (2015) found that mathematics anxiety can even occur before the exposure to mathematics activities, which then interferes with learning mathematical materials in the classroom.

## The Impact of Mathematics Anxiety on Mathematics Performance and Stem-Related Careers

Mathematics performance has been measured in many different ways using, for example, mathematics tests and Grade Point Average (GPA) (Hart and Ganley, 2019). Several studies have found that there is a negative relationship between mathematics anxiety and mathematics performance (Ma, 1999; Hart and Ganley, 2019; Namkung et al., 2019; Zhang et al., 2019). Furthermore, mathematics anxiety was also found to impact STEM-related attitudes and careers. According to Tsupros et al. (2009), “*STEM education is an interdisciplinary approach to learning where rigorous academic concepts are coupled with real-world lessons as students apply science, technology, engineering, and math in contexts that make connections between school, community, work, and the global enterprise enabling the development of STEM literacy and with it the ability to compete in the new economy*.” According to Brown et al. (2011), STEM education has been defined as “*a standards-based, meta-discipline residing at the school level where all teachers, especially science, technology, engineering, and math (STEM) teachers, teach an integrated approach to teaching and learning, where discipline-specific content is not divided, but addressed and treated as one lively, fluid study*.” In an epidemiological study conducted in the US using 3,000 students, Ahmed (2018) found that mathematics anxiety was related to the avoidance of STEM career choices. Further, several studies have shown that mathematics anxiety impacts successful education and future employment (Hembree, 1990; Ashcraft and Krause, 2007).

## Factors Underlying Mathematics Anxiety

Students may develop mathematics anxiety for different reasons (Hartwright et al., 2018; McDonough and Ramirez, 2018). For example, some students may develop mathematics anxiety due to having general trait anxiety (Baloglu, 1999; Kazelskis et al., 2000; Cipora et al., 2015; O'Leary et al., 2017; Paechter et al., 2017; Lauer et al., 2018), which is a relatively a stable individual disposition and related to feeling anxiety across different situations and in different environments. Students with high trait anxiety may experience anxiety upon exposure to complex mathematical problems.

General trait anxiety was found to be related to emotional dysregulation (Amstadter, 2008). Accordingly, individual differences in emotional regulation were also related to the development of mathematics anxiety (Klein et al., 2019). Further, some studies have shown that emotional dysregulation is related to low mathematics performance and mathematics anxiety (Brunyé et al., 2013; Artemenko et al., 2015; Pizzie and Kraemer, 2018; Klein et al., 2019). The link between emotional dysregulation and mathematics anxiety can be explained as follows: Mathematics anxiety is related to an increase in emotional feelings. However, a weak emotional regulation mechanism cannot provide a cognitive control system to manage such intense feelings, leading to the expression of mathematics anxiety (for discussion see Turk et al., 2005; Suveg et al., 2018).

In addition, mathematics anxiety in some individuals may be related to negative mathematics beliefs, that is, beliefs about one's mathematics capabilities (e.g., girls are not good at math, I am not a math person, math is so confusing, or math is scary, Bieg et al., 2015; Buckley et al., 2016; Ramirez et al., 2018). Along these lines, studies found that low self-esteem (Das et al., 2014; Jameson and Fusco, 2014; Balmeo and Fabella, 2018) and low self-efficacy (i.e., belief in one's ability to succeed, Walsh, 2008; Russo et al., 2014) are related to increased mathematics anxiety and reduced mathematics performance. This is probably related to students' beliefs on their inability to understand or succeed in mathematics courses. It is unclear how negative mathematics beliefs could be related to mathematics anxiety and mathematics performance. However, one explanation could be that metacognitive processes mediate the relationship between negative mathematics beliefs and mathematics performance, as reported in several studies (for discussion see Legg and Locker Jr, 2009; Filippello et al., 2016; Buzzai et al., 2020; Gabriel et al., 2020). Future research should investigate the exact mechanism of how beliefs, metacognition, and mathematics performance interact.

The development of mathematics anxiety could also stem from interactions with parents and teachers (Berkowitz et al., 2015; Maloney et al., 2015). For example, parental pressure was found to negatively impact students' mathematics learning and mathematics anxiety (Putwain and Symes, 2011). Along these lines, one recent study found that mathematics anxiety is related to authoritarian parenting style (Macmull and Ashkenazi, 2019). In addition, some studies found that teachers' use of fear appeals (e.g., teachers telling students “work hard or you will fail”) may also lead to anxiety in students (Putwain and Roberts, 2009; Putwain and Symes, 2011).

## Developing an Intervention for Mathematics Anxiety

Until now, most schools use tutoring programs to manage mathematics anxiety and low mathematics performance. For example, Supekar et al. (2015) presented a sample of 46 grade 3 students to an intensive 8 week one-to-one tutoring program (consisted of 22 lessons of increasing difficulty; lasting 15–20 h in total). Supekar et al. (2015) found that tutoring remediated aberrant functional responses and connectivity in emotion-related neural circuits including the basolateral amygdala. However, in this study, the decrease in mathematics anxiety following tutoring was modest. Generally speaking, tutoring is not individualized and does not target emotional and cognitive issues associated with mathematics anxiety.

There are few existing psychological intervention studies that aimed to reduce mathematics anxiety such as reappraising situations (Johns et al., 2008; Jamieson et al., 2010; Pizzie et al., 2020), expressive writing (Ramirez and Beilock, 2011; Park et al., 2014), and focused relaxation (Brunyé et al., 2013). However, these methods do not address the reasons and nature of mathematics anxiety in different individuals.

## A Novel Individualized Cognitive Behavioral Therapy (i-CBT) for Mathematics Anxiety

Given its important relationship to mathematics performance and interest in STEM careers and that different individuals develop mathematics anxiety due to different reasons, it is important to targets the reasons underlying the development of this form of anxiety in different individuals. Along these lines, on March 13, 2008, the National Mathematics Advisory Panel presented “Foundations for Success: The Final Report of the National Mathematics Advisory Panel” to the President of the United States and the Secretary of Education (U.S. Department of Education). This report concluded that “*anxiety about math performance is related to low math grades, failure to enroll in advanced math courses, and poor scores on standardized tests of math achievement*.” Accordingly, this report strongly recommended “*development of promising interventions for reducing serious math anxiety*.” To our surprise, this is not the case yet.

Prior studies have shown CBT may be effective at reducing anxiety in general (Orbach et al., 2007; Bandelow et al., 2015; Tay et al., 2019). To our knowledge, there is only one study on the use of group CBT to effectively reduce mathematics anxiety in India (Karimi and Venkatesan, 2009). Being a group therapy, this study did not address individual differences among the participants. Here, we argue that clinicians and researchers should develop an individualized Cognitive Behavioral Therapy (i-CBT) intervention to reduce mathematics anxiety, which could, in turn, increase mathematics performance, and enhance STEM-career interests and attitudes. Below, we explain how the i-CBT can be used to ameliorate the individual factors that have led to mathematics anxiety in students.

Initially, students should undergo an initial testing to determine factors related to their mathematics anxiety, as discussed in the section above. It is expected that some students may show mathematics anxiety due to their belief, low self-esteem, or to general trait anxiety. Subsequently, if the initial screening phase show that some students suffer from general trait anxiety, then their i-CBT should include sessions that target trait anxiety. In addition, relaxation could reduce general trait anxiety as well as mathematics anxiety. Brunyé et al. (2013) divided a sample of undergraduate university students to high vs. low mathematics-anxious groups using the 30-item Mathematics Anxiety Rating Scale. Each group was given three breathing exercises, each lasting for 15 mins using relaxation-recording instructions. These exercises included focused breathing (attentional focusing on the positive sensations of the breath), unfocused breathing (thinking freely without trying to focus on anything in particular), and worry exercise (answering silently a series of anxiety-inducing questions). The results showed that focused breathing improved the accuracy of performing a mental arithmetic task and increased calmness in high but not in low mathematics-anxious group.

Individuals with emotional dysregulation due to mathematics anxiety will benefit from a practice that involves a reappraisal of their anxiety. For example, Johns et al. (2008) found that instructing participants to reappraise the anxiety they felt about mathematics led to a better mathematics performance than in a control condition. Therefore, changing emotion regulation strategies could improve mathematics anxiety and performance in individuals with emotional dysregulation. Recent studies provided support for using emotion regulation therapy with general anxiety disorders (e.g., see Mennin et al., 2018). Therefore, i-CBT techniques may ameliorate maladaptive cognitive emotion regulation strategies, which in turn may reduce mathematics anxiety and improve mathematics performance in our students. Along these lines, i-CBT should also include sessions on cognitive reappraisal to manage mathematics anxiety, which is supported by recent studies (Pizzie and Kraemer, 2021). Furthermore, expressive writing may also reduce mathematics anxiety, as it may help understand reasons underlying this form of anxiety. While this assumption was not, to our knowledge, investigated, a recent study found that expressive writing can reduce test anxiety in students (Shen et al., 2018).

We argue that i-CBT program should include sessions on identifying and modifying wrong beliefs related to mathematics anxiety and issues related to low self-esteem and self-efficacy (e.g., “girls cannot do math” or “I am not a math person”), in individuals with erroneous beliefs about mathematics practices. Standard CBT often includes sessions on changing erroneous beliefs (Pittig et al., 2019), and exiting CBT interventions have also been used to increase self-esteem (Morton et al., 2012; Sonmez et al., 2020). Further, i-CBT should include sessions to target mathematics anxiety developed due to parental pressure and/or teachers' use of fear appeals. It is possible that parental pressure and teachers' use of fear appeals may increase trait anxiety in the students and perhaps impact their self-esteem. Here it is potentially important to include parents in treatment. Some research has shown that parents can assist their children recover from anxiety in general (Hirshfeld-Becker et al., 2019), although we are not aware of any family-based interventions for mathematics anxiety.

## Conclusions

In this opinion article, we show that (i) mathematics anxiety is quite common among different cultures, (ii) mathematics anxiety impacts mathematics performance and STEM careers and attitudes, and (iii) different students develop mathematics anxiety due to different reasons. Accordingly, we argue that future i-CBT program should aim to change negative affective and cognitive patterns to minimize mathematics anxiety in students. A future i-CBT intervention should aim at (i) identifying factors underlying mathematics anxiety in different students and (ii) incorporating different sessions that target factors leading to mathematics anxiety in different individuals. One benefit of individualized treatment is that it can be conducted online and can thus target a large number of students living in both urban and rural areas.

## Author Contributions

All authors listed have made a substantial, direct and intellectual contribution to the work, and approved it for publication.

## Funding

This study was supported in the form of funding by NPRP-C # Subproject (NPRP12C-33955-SP-90), which is part of a cluster project (NPRP12C-0828-190023), and from the Qatar National Research Fund (a member of Qatar foundation). The statements made herein are solely the responsibility of the authors.

## Conflict of Interest

The authors declare that the research was conducted in the absence of any commercial or financial relationships that could be construed as a potential conflict of interest.

## Publisher's Note

All claims expressed in this article are solely those of the authors and do not necessarily represent those of their affiliated organizations, or those of the publisher, the editors and the reviewers. Any product that may be evaluated in this article, or claim that may be made by its manufacturer, is not guaranteed or endorsed by the publisher.
